# Paralysis Not Easily Caught

**Published:** 1916-08

**Authors:** Simon Flexner

**Affiliations:** Director of Laboratories of the Rockefeller Institute for Medical Research


### Paralysis Not Easily Caught.

BY SIMON FLEXNER, M. D., DIRECTOR OF LABORATORIES OF THE ROCKEFELLER INSTITUTE FOR MEDICAL RESEARCH.

The Rockefeller Institute for Medical Research has been appealed to by so many physicians and laymen for information and advice on the subject of infantile paralysis that it has seemed desirable to relate the facts of present knowledge concerning certain highly pertinent aspects of the disease, together with deductions of practical importance derived from them.

### NATURE.

Infantile paralysis is an infectious and communicable disease which is caused by the invasion of the central nervous organs—the spinal cord and brain—by a minute, filterable micro-organism which has now been secured in artificial culture and as such is distinctly visible under the higher powers of the microscope.

The virus of infantile paralysis, as the micro-organism causing it is termed, exits constantly in the central nervous organs and upon the mucous membrane of the nose and throat and of the intestines in persons suffering from the disease; it occurs less frequently in the other internal organs, and it has not been detected in the general circulating blood of patients.

### LOCATION OF VIRUS IN HEALTHY PERSONS.

Although the micro-organism of infantile paralysis is now known, the difficulties attending its artificial cultivation and identification under the microscope are such as to make futile the employment of ordinary bacteriological tests for its detection. Nevertheless, the virus can be detected by inoculation tests upon monkeys, which animals develop a disease corresponding to infantile paralysis in human beings. In this manner the fact has been determined that the mucous membrane of the nose and throat of healthy persons who have been in intimate contact with acute cases of infantile paralysis may become contaminated with the virus, and that such contaminated persons without falling ill themselves may convey the infection to other persons, chiefly children, who develop the disease.

### RELATION OF VIRUS TO TYPES OF THE DISEASE.

The virus has, apparently, an identical distribution irrespective of the types or severity of cases of infantile paralysis. Whether the cases correspond with the so-called abortive forms of the disease in which definite paralysis of the muscles does not occur at all, or is so slight and fleeting as often to escape detection; whether they correspond with the meningeal forms in which the symptoms resemble those of acute meningitis, with which muscular paralysis may or may not be associated; or whether they consist of the familiar paralytic condition, the virus is present not only within the nervous organs but also upon the mucous membranes of the nose, throat and intestines.

### ESCAPE OF THE VIRUS FROM THE BODY.

Micro-organisms which convey disease escape from the body of an infected individual in a manner enabling them to enter and multiply within fresh or uninfected individuals in such a manner as to cause further disease. The virus of infantile paralysis is known to leave the infected human body in the secretions of the nose, throat and intestines. It also escapes from contaminated healthy persons in the secretions of the nose and throat. Whether it ever leaves the infected body in other ways is unknown. At one time certain experiments seemed to show that biting insects, and particularly the stable fly, might withdraw the virus from the blood of infected persons and inoculate it into the blood of healthy persons. But as the virus has never been detected in the blood of human beings and later experiments with the stable fly have not confirmed the earlier ones, this means of escape of the virus must be considered doubtful. On the other hand, it has been shown by experiments on animals, so that the same facts should be regarded as applicable to human beings, that the virus seeks to escape from the body by way of the nose and throat, not only when inoculation takes place through these membranes, but also when the inoculation is experimentally made into the abdominal cavity, the blood or the brain itself. From this it is concluded that the usual means of escape of the virus is by way of the ordinary secretions of the nose and throat, and, after swallowing these, with the discharges of the intestines.

### ENTRANCE OF THE VIRUS INTO THE BODY.

The virus enters the body, as a rule if not exclusively, by way of the mucous membrane of the nose and throat. Having gained entrance to those easily accessible parts of the body, multiplication of the virus occurs there, after which it penetrates to the brain and spinal cord by way of the lymphatic channels which connect the upper nasal membrane with the interior of the skull.

Whether the virus ever enters the body in any other way is unknown. Certain experiments already alluded to make it possible that it may be inoculated into the blood by insects, and other experiments have shown that, under peculiar and extraordinary conditions, it may in monkeys enter through the intestines. But, while the latter two modes of infection may operate sometimes, observations upon human cases of infantile paralysis and upon animals all indicate that the main avenue of entrance of the virus into the body is by way of upper respiratory mucous membrane—that is, the membrane of the nose and throat.

### RESISTANCE OF THE VIRUS.

The physical properties of the virus of infantile paralysis adapt it well for conveyance to the nose and throat. Being contained in their secretions, it is readily distributed by coughing, sneezing, kissing, and by means of fingers and articles contaminated with these secretions, as well as with the intestinal discharges. Moreover, as the virus is thrown off from the body mingled with the secretions, it withstands for a long time even the highest summer temperatures, complete drying, and even the action of weak chemicals, such as glycerine and carbolic acid, which destroy ordinary bacteria. Hence mere drying of the secretions is no protection; on the contrary, as the dried secretions may be converted into dust which is breathed into the nose and throat, they become a potential source of infection. The survival of the virus in the secretions is favored by weak daylight and darkness, and hindered by bright daylight and sunshine. It is readily destroyed by exposure to sunlight.

### CONVEYANCE BY INSECTS.

Since epidemics of infantile paralysis always arise during the period of warm or summer weather, they have been thought of as possibly being connected with or dependent on insect life. The blood-sucking insects have especially come under suspicion. Experiments have been made with biting flies, bed bugs, mosquitoes and with lice. Neither mosquitoes nor lice seem able to take the virus from the blood of infected monkeys or to retain it for a time in a living state. In one instance, bed bugs have been made to take up the virus from the blood of monkeys, but they did not convey it by biting to healthy monkeys. Certain experiments did indicate that the biting stable fly could both withdraw the virus from the blood of infected and reconvey it to the blood of healthy monkeys, which became paralyzed. But more recent studies have failed to confirm the earlier ones. Moreover, experimentally inoculated monkeys differ in one way from human beings suffering from infantile paralysis, for while the virus may appear in the blood of the former, it has never been detected in the blood of the latter. The ordinary or domestic fly may become contaminated with the virus contained in the secretions of the body and serve as the agent of its transportation to persons and to food with which they come into contact. Domestic flies experimentally contaminated with the virus remain infective for forty-eight hours or longer. While our present knowledge excludes insects from being active agents in the dissemination of infantile paralysis, they nevertheless fall under suspicion as being potential mechanical carriers of the virus of that disease.

### CONVEYANCE BY DOMESTIC ANIMALS.

The attention which the recent epidemic of infantile paralysis has drawn to the diseases attended by paralysis has led to the discovery that domestic animals and pets are subject to paralytic diseases. The animals which have especially come under suspicion as possibly distributing the germ of infantile paralysis are poultry, pigs and dogs and cats. But in isolated instances, sheep, cattle and even horses have been suspected. All these kinds of animals are subject to diseases in which paralysis of the legs and other parts of the body sometimes appears. In not a few instances paralytic diseases among poultry or pigs have been noted to coincide with the appearance of cases of infantile paralysis on a farm or in a community. Experimental studies have, however, excluded the above mentioned animals from being carriers of the virus of infantile paralysis. The paralytic diseases which they suffer have long been known and are quite different from infantile paralysis. Their occurrence may be coincidental; in no instance investigated has one been found to be responsible for the other.

### ROUTES OF TRAVEL.

Studies carried out in various countries in which infantile paralysis has been epidemic all indicate that, in extending from place to place or point to point, the route taken is that of ordinary travel. This is equally true whether the route is by water or land, along a simple highway or the line of a railroad. In other words, the evidence derived from this class of studies confirms the evidence obtained from other sources in connecting the distributing agency intimately with human beings and their activities.

### SURVIVAL OF THE VIRUS IN THE INFECTED BODY.

The virus of infantile paralysis is destroyed in the interior of the body more quickly and completely than, in some instances, in the mucous membrane of the nose and throat. It has been found in monkeys, in which accurate experiments can be carried out, that the virus may disappear from the brain and spinal cord within a few days to three weeks after the appearance of the paralysis, while at the same time it is still present upon the mucous membranes mentioned. The longest period after inoculation in which the virus has been detected in the mucous membrane of the nose and throat of monkeys is six months. It is far more difficult to detect the human than the money carriers of the virus since, as directly obtained from human beings, the virus displays a low degree of infectivity for monkeys; while once adapted to monkeys, the virus becomes incredibly active, so that minute quantities are capable of ready detection by inoculation tests. Yet in an undoubted instance of the human disease the virus was detected in the mucous membrane of the throat five months after its acute onset. Hence, we possess conclusive evidence of the occurrence of occasional chronic human carriers of the virus of infantile paralysis.

### FLUCTUATION IN EPIDEMICS.

Not all epidemics of infantile paralysis are equally severe. Indeed, great variations or fluctuations are known to occur not only in the number of cases, but also in the death rate. The extremes are represented by the occasional instances of infantile paralysis known in every considerable community and from which no extension takes place, and the instances in which in a few days or weeks the number of cases rises by leaps and bounds into the hundreds and the death rate reaches 20 per cent or more of those attacked. While the factors which determine this discrepancy are not known, certain of them have become apparent. A factor of high importance is the infective power or potency, or, technically stated, the virulence, of the micro-organism or virus causing the disease. This virus is subject to fluctuations of intensity which can best be illustrated by an example. The virus as ordinarily present in human beings even during severe epidemics has low infective power for monkeys. But by passing it from monkey to monkey it tends to acquire, after a variable number of such passages, an incredible activity. However, occasional samples of the human virus refuse to be thus intensified. But once rendered highly potent, the virus may be passed from monkey to monkey through a long but not indefinite series. Finally, in some samples of the virus at least a reverse change takes place—the virus begins to lose its virulence until it returns to the original or even to a diminished degree of infective power. In this respect the behavior of the virus corresponds to the onset, rise and then the fall in number and severity of cases as observed in the course of epidemics of infantile paralysis and other epidemic diseases. Hence either a new active specimen of the virus may be introduced from without which, after a certain number of passages from person to person, acquires a high potency; or a specimen of virus already present and left over from a previous epidemic after a resting period and similar passages again becomes active and reaches an infective power which equals or even exceeds that originally possessed. Another but more indefinite factor relates to the degree of susceptibility among children and others affected which at one period may be greater or less than at another.

VARYING INDIVIDUAL SUSCEPTIBILITIES.

Not all children and relatively few adults are susceptible to infantile paralysis. Young children are more susceptible, generally speaking, than older ones; but no age can be said to be absolutely insusceptible. When several children exist in a family or in a group, one or more may be affected, while the others escape or seem to escape. The closer the family or other groups are studied by physicians the more numerous it now appears are the number of cases among them. This means that the term infantile paralysis is a misnomer, since the disease arises without causing any paralysis whatever, or such slight and fleeting paralysis as to be difficult of detection. The light or abortive cases, as they are called, indicate a greater general susceptibility than has always been recognized, and their discovery promises to have far-reaching consequences in respect to the means employed to limit the spread or eradicate foci of the disease.

PERIOD OF INCUBATION.

Like all other infectious diseases, infantile paralysis does not arise at once after exposure, but only after an intervening lapse of time called the period of incubation. This period is subject to wide limits of fluctuation. In certain instances it has been as short as two days, in others it has been two weeks or possibly even longer. But the usual period does not exceed about eight days.

### PERIOD OF INFECTIVITY.

Probably the period at which the danger of communication is greatest is during the very early and acuter stage of the disease. This statement must be made tentatively since it depends on inference, based on general knowledge of infection rather than on demonstration. Judging from experiments on animals, the virus tends not to persist in the body longer than four or five weeks except in those exceptional instances in which chronic carriage is developed. Hence cases of infantile paralysis which have been kept under supervision for a period of six weeks from the onset of the symptoms may be regarded as practically free of danger.

### PROTECTION BY PREVIOUS ATTACK.

Infantile paralysis is one of the infectious diseases in which insusceptibility is conferred by one attack. The evidence derived from experiments on monkeys is conclusive in showing that an infection which ends in recovery gives protection from a subsequent inoculation. Observations upon human beings have brought out the same fact, which appears to be generally true, and to include all the forms of infantile paralysis, namely the paralytic, meningeal, or abortive, which all confer immunity.

### BASIS OF THE IMMUNITY.

The blood of normal persons and monkeys is not capable of destroying or neutralizing the effect of the virus of infantile paralysis. The blood of persons or monkeys who have recovered from the disease is capable of destroying or neutralizing the effect of the virus. The insusceptibility or immunity to subsequent infection, whether occurring in human beings after exposure or monkeys after inoculation, rests on the presence of the destroying substances, the so-called immunity bodies, which arise in the internal organs and are yielded to the blood. So long as these immunity bodies persist in the body, protection is afforded; and their presence has been detected twenty years or even longer after recovery from infantile paralysis. Experiments have shown that the immunity bodies appear in the blood in the course of even the mildest attack of the disease, which fact explains why protection is afforded irrespective of the severity of the case.

### ACTIVE IMMUNIZATION.

Protection has been afforded monkeys against inoculation with effective quantities of the virus of infantile paralysis by previously subjecting them to inoculation with sub-effective quantities or doses of the virus. By this means and without any evident illness or effect of the protective inoculation, complete immunity has been achieved. But the method is not perfect since in certain instances not only was immunity not obtained, but unexpected paralysis intervened. In the instances in which protection was accomplished, the immunity bodies appeared in the blood.

### PASSIVE PROTECTION.

By transferring the blood of immune monkeys to normal or untreated ones, they can be rendered insusceptible or immune, and the immunity will endure for a relatively short period during which the passively transferred immunity bodies persist. The accomplishment of passive immunization is somewhat uncertain, and its brief duration renders it useless for purposes of protective immunization.

### SERUM TREATMENT.

On the other hand, a measure of success has been achieved in the experimental serum treatment of inoculated monkeys. For this purpose blood serum derived either from recovered and protected monkeys or human beings has been employed. The serum is injected into the membranes about the spinal cord, and the virus is inoculated into the brain. The injection of serum must be repeated several times in order to be effective. Use of this method has been made in a few instances in France, where the blood serum derived from persons who had recovered from infantile paralysis has been injected into the spinal membranes of persons who have just become paralyzed. The results are said to be promising. Unfortunately, the quantity of the human immune serum is very limited, and no other animals than monkeys seem capable of yielding an immune serum and the monkey is not a practicable animal from which to obtain supplies.

### DRUG TREATMENT.

The virus of infantile paralysis attacks and attaches itself to the central nervous organs. Hence it is reached not only with difficulty because nature has carefully protected those sensitive organs from injurious materials which may gain access to the blood, but it must be counteracted by substances and in a manner that will not themselves injure those sensitive parts. The ideal means to accomplish this purpose is through the employment of an immune serum, since serums are among the least injurious therapeutic agents. The only drug which has shown any useful degree of activity is hexamethylenamin, which is itself germicidal, and has the merit of entering the membranes, as well as the substance of the spinal cord and brain in which the virus is deposited. But experiments on monkeys have shown this chemical to be effective only very early in the course of the inoculation and only in a part of the animals treated. Efforts to modify and improve this drug by chemical means have up to the present time been only partially successful. The experiments have not yet reached the point where the new drugs are applicable to the treatment of human cases of infantile paralysis.

### PRACTICAL DEDUCTIONS AND APPLICATIONS.

1. The chief mode of demonstrated conveyance of the virus is through the agency of human beings. Whether still other modes of dissemination exist is unknown. According to our present knowledge, the virus leaves the body in the secretions of the nose and throat and in the discharges from the intestines. The conveyors of the virus include persons ill of infantile paralysis in any of its several forms and irrespective of whether they are paralyzed or not, and such healthy persons who may have become contaminated by attendance on or association with the ill. How numerous the latter class may be is unknown. But all attendants on or associates of the sick are suspect. These healthy carriers rarely themselves fall ill of the disease; they may, however, be the source of infection in others. On the other hand, the fact that infantile paralysis is very rarely communicated in general hospitals to other persons, whether doctors, nurses or patients, indicates that its spread is subject to ready control under restricted and supervised sanitary conditions.

2. The chief means by which the secretions of the nose and throat are disseminated is through the act of kissing, coughing or sneezing. Hence, during the prevalence of an epidemic of infantile paralysis, care should be exercised to restrict the distribution as far as possible through these common means. Habits of self-denial, care and cleanliness and consideration for the public welfare can be made to go very far in limiting the dangers from these sources.

Moreover, since the disease attacks by preference young children and infants, in whom the secretions from the nose and mouth are wiped away by mother or nurse, the fingers of these persons readily become contaminated. Through attentions on other children or the preparation of food which may be contaminated, the virus may thus be conveyed from the sick to the healthy. The conditions which obtain in a household in which a mother waits on the sick child and attends the other children are directly contrasted with those existing in a well ordered hospital; the one is a menace, the other a protection to the community. Moreover, in homes the practice of carrying small children about and comforting them is the rule, through which not only the hands, but other parts of the body and the clothing of parents may become contaminated.

3. Flies also often collect about the nose and mouth of patients ill of infantile paralysis and feed on the secretions, and they even gain access to the discharges from the intestines in homes unprotected by screens. This fact relates to the domestic fly, which becoming grossly contaminated with the virus, may deposit it on the nose and mouth of healthy persons, or upon food or eating utensils. To what extent the biting stable fly is to be incriminated as a carrier of infection is doubtful; but we already know enough to wish to exclude from the sick, and hence from menacing the well, all objectionable household insects.

Food exposed to sale may become contaminated by flies or from fingers which have been in contact with secretions containing the virus; hence food should not be exposed in shops and no person in attendance upon a case of infantile paralysis should be permitted to handle food for sale to the general public.

4. Protection to the public can be best secured through the discovery and isolation of those ill of the disease, and the sanitary control of those persons who have associated with the sick and whose business calls them away from home. Both these conditions can be secured without too great interference with the comforts and the rights of individuals.

In the first place where homes are not suited to the care of the ill so that other children in the same or adjacent families are exposed, the parent should consent to removal to hospital in the interest of the sick child itself, as well as in the interest of other children. But this removal or care must include not only the frankly paralyzed cases, but also the other forms of the disease. In the event of doubtful diagnosis, the aid of the laboratory is to be sought since even in the mildest cases changes will be detected in the cerebro-spinal fluid removed by lumbar puncture. If the effort is to be made to control the disease by isolation and segregation of the ill, then these means must be made as inclusive as possible. It is obvious that in certain homes isolation can be carried out as effectively as in hospitals.

PERSONAL CARE SAVES.

But what has been said of the small incidence of cases of the disease among the hospital personnel and those with whom they come into contact, indicates the extent to which personal care of the body by adults and responsible people can diminish the menace which those accidentally or unavoidably in contact with the ill are to the community. Care exercised not to scatter the secretions of the nose and throat by spitting, coughing and sneezing, the free use of clean handkerchiefs, cleanliness in habits affecting especially the hands and face, changes of clothes, etc., should all serve to diminish this danger.

In the end the early detection and isolation of the cases of infantile paralysis in all of its forms, with the attendant control of the households from which they come, will have to be relied upon as the chief measure of staying the progress of the epidemic.

5. The degree of susceptibility of children and other members of the community to infantile paralysis is relatively small, and is definitely lower than to such communicable diseases as measles, scarlet fever and diphtheria. This fact in itself constitutes a measure of control; and while it does not justify the abatement of any practicable means which may be employed to limit and suppress the epidemic, it should tend to prevent a state of over-anxiety and panic from taking hold of the community.

6. A percentage of persons, children particularly, die during the acute stage of the disease. This percentage varies from five in certain severe epidemics to twenty in others. The average death rate of many epidemics has been below 10 per cent. A reported high death rate may not be actual, but only apparent, since in every instance the death will be recorded, while many cases which recover may not be reported at all to the authorities. In the present instance it is too early in the course of the epidemic to calculate the death rate, which may prove to be considerably lower than it now seems to be.

7. Of those who survive, a part make complete recoveries, in which no crippling whatever remains. This number is greater than is usually supposed, because it includes not only the relatively large number of slight or abortive cases, but also a considerable number of cases in which more or less of paralysis was present at one time. The disappearance of the paralysis may be rapid or gradual—may be complete in a few days or may require several weeks or months.

CRIPPLED PERMANENTLY.

The remainder, and, unfortunately, not a small number, suffer some degree of permanent crippling. But even in this class, the extent to which recovery from the paralysis may occur is very great. In many instances the residue of paralysis may be so small as not seriously to hamper the life activities of the individual; in others in whom it is greater it may be relieved or minimized by suitable orthopedic treatment. But what it is imperative to keep in mind is that the recovery of paralyzed parts and the restoration of lost muscular power and function is a process which extends over a long period of time—that is, over months and even years. So that even a severely paralyzed child who has made little recovery of function by the time the acute stage of the disease is over, may go on gaining for weeks, months, and even years, until in the end he has regained a large part of his losses. Fortunately, only a very small number of the attacked are left severely and helplessly crippled. Lamentable as it is that even one should be so affected, it is, nevertheless, a reassurance to know that so many recover altogether and so much of what appears to be permanent paralysis disappears in time.

There exists at present no safe method of preventive inoculation or vaccination and no practicable method of specific treatment. The prevention of the disease must be accomplished through general sanitary means; recovery from the disease is a spontaneous process which can be greatly assisted by proper medical and surgical care. Infantile paralysis is an infectious disease, due to a definite and specific micro-organism or virus; recovery is accomplished by a process of immunization which takes place during the acute period of the disease. The tendency of the disease is toward recovery, and it is chiefly or only because the paralysis in some instances involves those portions of the brain and spinal cord which control respiration or breathing and the heart's action that death results.

Finally, it should be added that not since 1907, at which time the great epidemic of infantile paralysis, or poliomyelitis, appeared in this country, has the country or this State or city been free of the disease. Each summer since has seen some degree of accession in the number of the cases; the rapid rise in the number of cases this year probably exceeds that of any previous year. But it must be remembered that in 1908 several thousand cases occurred in the greater city—possibly, indeed, many cases of and deaths due to the disease were never reported as such. Hence, the present experience, severe and serious as it is, is not something new; the disease has been severely epidemic before and was brought under control. The knowledge regarding it now is far greater than it was in 1908; and the forces of the city which are dealing with the epidemic are probably better organized and in more general co-operation than ever before. The outlook, therefore, should not be regarded as discouraging.—*New York Tribune.*

